# Evaluation of changes in price, volume and expenditure of PD-1 drugs following the government reimbursement negotiation in China: a multiple-treatment period interrupted time series analysis

**DOI:** 10.7189/jogh.15.04069

**Published:** 2025-04-18

**Authors:** Hongbin Yi, Mengtian Cai, Xiaoxia Wei, Yingdan Cao, Liping Kuai, Dongyan Xu, Yue Qiu, Sheng Han

**Affiliations:** 1 Department of Pharmacy Administration and Clinical Pharmacy, School of Pharmaceutical Sciences, Peking University, Beijing, China; 2 International Research Centre for Medicinal Administration, Peking University, Beijing, China; 3 Department of Pharmacy, Shengli Clinical Medical College of Fujian Medical University, Fujian Provincial Hospital, Fuzhou, China; 4 Science and Technology Development Centre, Chinese Pharmaceutical Association, Beijing, China; 5 Institute for Hospital Management, Tsinghua University, Beijing, China

## Abstract

**Background:**

Government reimbursement negotiation (GRN) is an important policy tool to increase the accessibility of drugs. In China, the impact of GRN implementation on programmed death-1 (PD-1) drugs price, procurement volume, and expenditure is unknown.

**Methods:**

This study used a multiple-treatment period interrupted time series design covering the period from the first-time recorded in Chinese Medical Economic Information database to 2022 to examine changes in hospital procurement price, volume and expenditure of PD-1 drugs after the implementation of GRN in China. Data were obtained from 698 public hospitals of 30 provinces in China.

**Results:**

A total of four PD-1 drugs have been selected in the National Reimbursement Drug List via GRN between March 2019 and 2022. After the implementation of the first-time GRN, the prices of all PD-1 drugs decreased significantly, with Camrelizumab experiencing the largest reduction in price and the largest increase in volume and expenditure. The Camrelizumab’s price decreased by 1151.75 Chinese Yuan (CNY) (β_2_ = −1151.75; 95% confidence interval (CI) = −1254.534, 1048.96), volume increased by 159.549 thousand defined daily doses (β_2_ = 159.549; 95% CI = 119.12, 199.979) and expenditure increased by 11.172 million CNY (β_2_ = 11.172; 95% CI = 1.653, 20.692). Following the implementation of the second-time of GRN, Sintilimab showed the largest decrease in price, with price decreased by 164.099 CNY (β_4_ = −164.099; 95% CI = −171.867, 156.331), Tislelizumab had the largest increase in volume and expenditure, with the volume increased by 102.185 thousand defined daily doses (β_4_ = 102.185; 95% CI = 47.862, 156.509) and expenditure increased by 4.119 million CNY (β_4_ = 4.119; 95% CI = −3.808, 12.047).

**Conclusions:**

The GRN policy improved the accessibility and affordability of PD-1 drugs. Health insurance policy-makers need to consider the legitimate interests of PD-1 drug manufacturers while ensuring the sustainability of the basic health insurance fund.

Cancer is a global problem that lacks a global solution [1]. A study indicated that there were 19.3 million new cases of cancer and almost 10 million deaths from cancer in 2020. Cancer is a significant cause of morbidity and mortality worldwide, across all regions and at all levels of human development [2].

Harnessing the power of the body’s immune system for the fight against cancer has been one of the most remarkable success stories of the past decade. Programmed death 1 (PD-1) checkpoint inhibitors were among the first cancer immunotherapies to be approved, and they have transformed the landscape of oncology treatment, offering hope to patients as new options for hard-to-treat cancers [3]. The efficacy of immunotherapies targeting the PD-1 pathway in the treatment of various cancers has been certified. These findings support the essential role of PD-1 and programmed death ligand 1 (PD-L1) in immune suppression.

By 2022, 10 PD-1 drugs have been approved for marketing in China. These PD-1 drugs are Nivolumab, Pembrolizumab, Toripalimab, Sintilimab, Camrelizumab, Tislelizumab, Penpulimab, Zimberelimab, Serplulimab and Pucotenlimab. There is evident that PD-1 drugs provide Chinese patients with a great range of treatment options. However, the high price of targeted anticancer drugs has placed a significant economic burden on both patients and health insurance systems [4].

The health care authority can actually influence the price of a new drug because it partially or totally pays this price to the pharmaceutical firm when providing public health insurance coverage to its citizens [5]. A targeted bargaining strategy using arbitration techniques to help health care insurance better balance innovation and affordability. Government reimbursement negotiation (GRN) is an important policy tool to lower drug prices and improve health care affordability and accessibility for patients through price negotiations between the government and drug manufacturers. Since 2015, the Chinese government has implemented several GRNs and achieved positive results. The impact of China's implementation of GRN on drug use can inform decision-making for global health care policy-makers [6–8].

Despite the implementation of numerous initiatives aimed at the prevention and control of cancer, the disease remains a significant public health concern in China [9]. In recent years, there has been a concerted effort to improve the availability of new cancer drugs in China [10–15]. Since 2017, the Chinese government has conducted annual GRN, and winning negotiated drugs are included in the National Reimbursement Drug List (NRDL), which is the list used for reimbursement of basic medical insurance in China. In order to increase the number of indications for reimbursement, drug manufacturers conducted the second-time GRN with the National Healthcare Security Administration, which is the basic medical insurance administration in China and is primarily responsible for the development and implementation of GRN. By 2022, only four PD-1 drugs will be included in the NRDL. The four PD-1 drugs in NRDL have all conducted the second-time GRN, and three of them successfully passed the second GRN. Sintilimab successfully negotiated government reimbursement in November 2019 and December 2021, and began implementation of negotiated engagements in January 2020 and January 2022 [16,17]. Toripalimab and Tislelizumab were successfully negotiated government reimbursement in December 2020 and December 2021, and began implementation of negotiated engagements in March 2021 and January 2022 [17,18]. Camrelizumab was successfully negotiated for government reimbursement in December 2020 and began implementation of the negotiated agreement in March 2021 [17]. (Table S1–2 in the **Online Supplementary Document**) Therefore, evaluating the impact of GRN for sintilmab, Toripalimab, Tislelizumab and Camrelizumab is representative and universal.

However, what is the impact of GRN on the price, procurement volume, and expenditure of PD-1 drugs in China? The aim of this study is to use monthly procurement data of PD-1 drugs from hospitals, and conducted multiple-treatment period interrupted time series (ITS) analyses to evaluate the changes in prices, procurement volume and expenditure of PD-1 drugs following the implementation of the GRN in China.

## METHODS

### Study data and sample

The study used the Chinese Medical Economic Information (CMEI) database to collect the monthly price, volume, and procurement expenditure data of all winning negotiated PD-1 drugs by drug generic name. The CMEI is a national hospital drug information collection and statistical analysis platform, with more than 1500 hospital procurement data sets [19]. This study included a continuous sample of 689 hospitals from 30 provinces in mainland China (excluding Tibet). As this study only analysed procurement data and did not involve human participants, obtaining informed consent from patients and ethical approval were not required. This study follows the Strengthening the Reporting of Observational Studies in Epidemiology reporting guideline [20].

The data includes two or three time periods for four PD-1 drugs:

1. Toripalimab: 24 months before the first-time of negotiation (March 2019–February 2021), 10 months from the first-time of negotiation to the second-time of negotiation (March 2021–December 2021), 12 months after the second time of negotiation (January 2022–December 2022)

2. Sintilimab: 10 months before the first-time of negotiation (March 2019–December 2019), 24 months after the first-time of negotiation to the second time of negotiation (January 2020–December 2021), 12 months after the second time of negotiation (January 2022–December 2022)

3. Camrelizumab: 19 months from marketing to negotiation (August 2019–February 2021) and 22 months after negotiation (March 2021–December 2022)

4. Tislelizumab: 12 months before the first-time of negotiation (March 2020–February 2021), 10 months from the first-time of negotiation to the second-time of negotiation (March 2021–December 2021), 12 months after the second-time of negotiation (January 2022–December 2022) (Table S1 in the **Online Supplementary Document**).

### Outcome measures

The objective of this study was to assess three outcome measures: price, hospital procurement volumes and expenditures. We use cost per defined daily dose (DDDc) and defined daily doses (DDDs) for PD-1 drugs to represent price and volume. The DDDc of each PD-1 drug was treated as a surrogate measure of the actual medication price paid, and the value is calculated as the ratio of procurement expenditure to volume procured. The DDDs recommended by the World Health Organization (WHO) for drug utilisation monitoring and research as a measure of purchase volume [21,22]. The DDDs were the number of daily doses of each PD-1 drug based on dosage regimens recommended in the product labels approved by the National Medical Products Administration [23].

### Statistical analysis

Both ITS and difference-in-differences are commonly used methods for policy evaluation. Difference-in-differences is not applicable to this study because there is no suitable control group and individual data. In addition, this study also wanted to observe the impact of GRN policy on PD-1 drug trends, so multiple-treatment period ITS analyses were more appropriate assessment models for this study [24,25]. The formula of multiple-treatment period ITS model is as follows:

*Y*_t_ *= β*_0_ *+ β*_1_*T*_t_ *+ β*_2_*X*1_t_ *+ β*_3_*X*1_t_*T*_t_ *+ β*_4_*X*2_t_ *+ β*_5_*X*2_t_*T*_t_ *+ ε*_t_

where *Y*_t_ is the summary outcome variable measured at each equally spaced time point t, *T*_t_ is the time since the start of the study (which can be years, quarters, or months), *X*_t_ is the dummy variable representing the intervention (pre-intervention period 0, otherwise 1), *X*_t_*T*_t_ is the interaction term, ε_t_ is the error term; *X*1_t_ represents the dummy variable of the first intervention, and *X*2_t_ represents the dummy variable of the second intervention; β_0_ represents the intercept or initial level of the outcome variable, β_1_ represents the slope or trend of the outcome variable before the first intervention, β_2_ represents the instantaneous level change after the first intervention, β_3_ represents the difference between the slope (trend) of the outcome before and after the first intervention, β_4_ represents the instantaneous level change after the second intervention and β_5_ represents the difference in outcome slope (trend) after the first intervention and after the second intervention.

β_2_ and β_4_ indicate changes in the level of PD-1 drugs at the time of GRN implementation. β_3_ and β_5_ indicate changes in the trend of PD-1 drugs before and after GRN implementation. Model estimates the coefficients by ordinary least-squares (OLS) regression producing Newey-West standard errors to handle autocorrelation in addition to possible heteroskedasticity [24]. A two-sided significance threshold was set at *P* < 0.05. The statistical analyses were conducted using Stata version 17.0 (StataCorp LLC, Texas, USA, 2019).

## RESULTS

### Changes in price of PD-1 drugs

**Figure 1:**
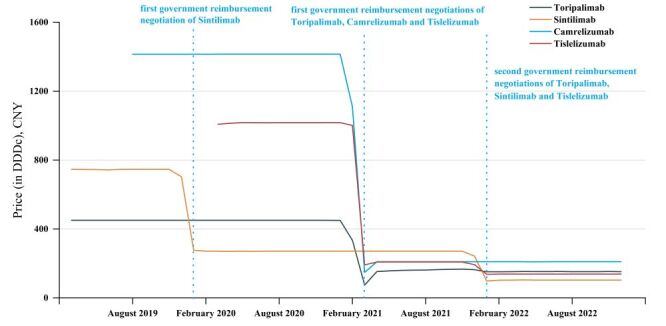
Prices of PD-1 drugs, first GRN implementation and second GRN implementation. CNY – Chinese Yuan, DDDc – cost per defined daily dose, GRN – government reimbursement negotiation, PD-1 – programmed death-1.

**Figure 1** shows that the price of PD-1 drugs decreased immediately following the first-time of GRN, and then continued to decrease following the second-time GRN. In contrast, the price of PD-1 drugs that did not participate GRN during the year remained stable. **Table 1** shows the price changes in the level and trend of four PD-1 drugs before and after GRN. After the implementation of the first-time of GRN, the Toripalimab’s price decreased by 304.415 CNY (β_2_ = −304.415; 95% confidence interval (CI) = −342.554, −266.276), the Sintilimab’s price decreased by 455.628 CNY (β_2_ = −455.628; 95% CI = −480.81, 430.447), the Camrelizumab’s price decreased by 1151.75 CNY (β_2_ = −1151.75; 95% CI = −1254.534, 1048.96), and the price of Tislelizumab was decreased by 809.9 CNY (β_2_ = −809.9; 95% CI = −820.686, −799.113). After the implementation of second-time GRN, the prices of three PD-1 drugs undergoing the second-time GRN decreased again. From the results of the trend change, only Toripalimab's trend was affected by the first-time of GRN (β_3_ = 6.988; 95% CI = −0.592, 14.569) (**Table 1**; Table S3 and Figure S1–4 in the **Online Supplementary Document**).

**Table1:** Changes in PD-1 drug prices, volumes and procurement expenditures before and after GRN implementation

Variable	Coefficient estimates (95% CI)			
	**First GRN**		**Second GRN**	
	**Change in level (β_2_)**	**Change in slope (β_3_)**	**Change in level (β_4_)**	**Change in slope (β_5_)**
**Price (in DDDc)**				
Toripalimab	−304.415 (−342.554, −266.276)*	6.988 (−0.592, 14.569)†	−32.964 (−59.487, −6.44)†	−5.735 (−12.055, 0.586)
Sintilimab	−455.628 (−480.81, −430.447)*	1.879 (−1.415, 5.174)	−164.099 (−171.867, −156.331)*	0.524 (−0.212, 1.259)
Camrelizumab	−1151.75 (−1254.534, −1048.96)*	5.52 (−3.478, 14.519)	NA	NA
Tislelizumab	−809.9 (−820.686, −799.113)*	0.226 (−2.57, 3.022)	−67.215 (−78.969, −55.461)*	−0.029 (−2.121, 2.063)
**Volume**				
Toripalimab	9.211 (−3.623, 22.045)	−0.27 (−1.715, 1.175)	5.282 (−5.737, 16.301)	2.847 (0.655, 5.038)†
Sintilimab	18.017 (3.214, 32.819)†	6.918 (5.847, 7.99)*	67.944 (10.856, 125.032)†	−2.363 (−12.226, 7.5)
Camrelizumab	159.549 (119.12, 199.979)*	−2.942 (−5.732, −0.151)†	NA	NA
Tislelizumab	23.837 (10.324, 37.35)‡	12.425 (8.54, 16.309)*	102.185 (47.862, 156.509)‡	0.027 (−10.458, 10.512)
**Expenditure**				
Toripalimab	−5.28 (−8.356, −2.204)‡	−0.061 (−0.404, 0.281)	−0.098 (−2.113, 1.916)	0.3 (−0.125, 0.725)
Sintilimab	2.595 (−1.867, 7.057)	1.514 (1.159, 1.87)*	−27.069 (−34.35, −19.789)*	−1.4 (−2.453, −0.348)†
Camrelizumab	11.172 (1.653, 20.692)†	−1.726 (−2.542, −0.909)*	NA	NA
Tislelizumab	2.345 (0.224, 4.466)†	2.241 (1.629, 2.853)*	4.119 (−3.808, 12.047)	−0.766 (−2.242, 0.71)

CI – confidence interval, DDDc – cost per defined daily dose, GRN – government reimbursement negotiation, NA – Not applicable

*P < 0.001.

†*P* < 0.05.

‡*P* < 0.01.

### Changes in volume of PD-1 drugs

**Figure 2:**
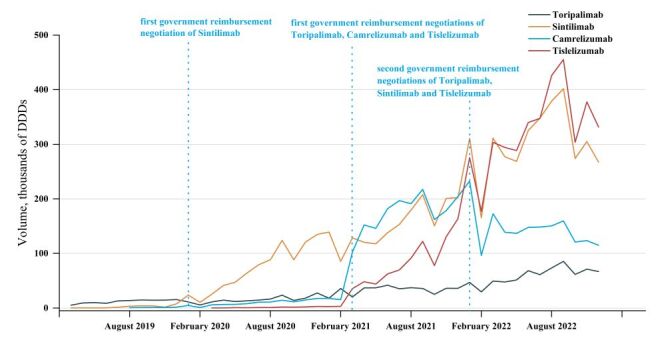
Volumes of PD-1 drugs, first GRN implementation and second GRN implementation. CNY – Chinese Yuan, DDDs – defined daily doses, GRN – government reimbursement negotiation, PD-1 – programmed death-1.

**Figure 2** shows that the volume of PD-1 drugs have an immediate increase after the first-time GRN and declined briefly after the second-time of GRN. However, the overall trend was upward. The second-time GRN of Camrelizumab was unsuccessful and therefore was not included in the NRDL. Consequently, the volume of Camrelizumab continued to decline.

After the implementation of first-time of GRN, the volume of Toripalimab increased by 9.211 thousand DDDs (β_2_ = 9.211; 95% CI = −3.623, 22.045), but the level change was not significant. Sintilimab's volume increased 18.017 thousand DDDs (β_2_ = 18.017; 95% CI = 3.214, 32.819), Camrelizumab’s volume increased 159.549 thousand DDDs (β_2_ = 159.549; 95% CI = 119.12, 199.979), and Tislelizumab’s volume increased 23.837 thousand DDDs (β_2_ = 23.837; 95% CI = 10.324, 37.35). The first-time GRN significantly affected the trends of volume for Sintilimab (β_3_ = 6.918; 95% CI = 5.847, 7.99), Camrelizumab (β_3_ = −2.942; 95% CI = −5.732, −0.151) and Tislelizumab (β_3_ = 12.425; 95% CI = 8.54, 16.309). After the second-time GRN, there was a significant change in the level of volume, although there was no significant change in the trend of volume for Sintilmab and Tislelizumab (**Table 1**; Table S4 and Figure S5–8 in the **Online Supplementary Document**).

### Changes in expenditure of PD-1 drugs

**Figure 3:**
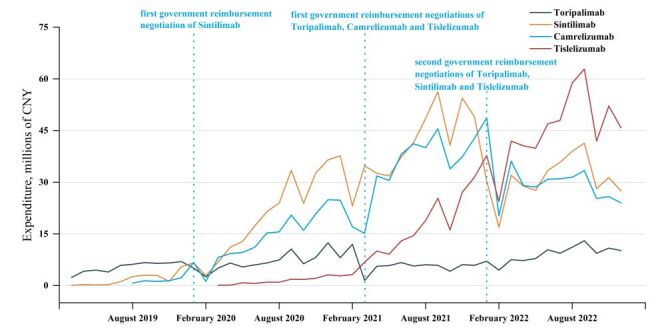
Expenditures of PD-1 drugs, first GRN implementation and second GRN implementation. CNY – Chinese Yuan, GRN – government reimbursement negotiation, PD-1 – programmed death-1.

**Figure 3** shows the changes in expenditure of PD-1 drugs. The procurement expenditures for PD-1 drugs increased immediately after the first GRN, and decreased briefly after the second GRN. However, there was a general upward trend in expenditure. Since the second-time GRN of Camrelizumab was unsuccessful, its procurement expenditure subsequently declined.

After the implementation of first-time GRN, the procurement expenditure of Toripalimab decreased by 5.28 million CNY (β_2_ = −5.28; 95% CI = −8.356, −2.204) in the month of policy implementation. The level of expenditure for Sintilimab did not change significantly. The procurement expenditure of Camrelizumab increased by 11.172 million CNY (β_2_ = 11.172; 95% CI = 1.653, 20.692) and Tislelizumab increased by 2.345 million CNY (β_2_ = 2.345; 95% CI, 0.224, 4.466). In addition to Toripalimab, the first-time GRN significantly affected Sintilimab (β_3_ = 1.514; 95% CI = 1.159, 1.87), Camrelizumab (β_3_ = −1.726; 95% CI = −2.542, −0.909) and Tislelizumab (β_3_ = 2.241; 95% CI = 1.629, 2.853) trends of procurement expenditure. The upward trends for Sintilimab and Tislelizumab were further enhanced. After the second-time GRN, only Sintilimab had a significant change in procurement expenditure (**Table 1**; Table S5 and Figure S9–12 in the **Online Supplementary Document**).

## DISCUSSION

Since the reform of drug approval in 2015, which introduced incentive policies, there has been a rapid increase in the number of Investigational New Drug (IND) and New Drug Application in China [26–28]. However, the PD-1 drug market is currently facing a number of challenges [29]. For patients and Medicare payers, the high price of PD-1 drugs is a heavy financial burden. In China, GRN policies are critical to improving affordability for patients [30]. Following implementation of the GRN, the prices of all PD-1 drugs decreased significantly, which is consistent with the results of previous studies [31–33]. While the long-term trend in PD-1 drug prices will not continue to decrease over time, the GRN resulted in a significant one-time decrease in PD-1 drug prices at the time of policy implementation and maintained the negotiated low prices over time. This change suggests that China's GRN policy has played a key role in reducing PD-1 drug prices and improving patient affordability. Although market competition and supply chain issues will influence drug prices [34,35], PD-1 drugs in this study are less influenced by these external factors. On the one hand, these PD-1 drugs are innovative drugs, most of the same drugs are still in the clinical trial stage, and the market competition is limited. On the other hand, as a key product of the manufacturer, the supply chain is strictly controlled, so the supply chain is relatively stable. In summary, for innovative drugs, in addition to increasing market competition and ensuring supply chain stability, the implementation of the GRN policy can also control the drug prices.

After PD-1 drugs are included in NRDL, the actual drug price paid by patients is lower than the market price because medicare covers part of the drug cost, the demand of the patient population will further increase. The results of this study show that the volume of the majority PD-1 drugs has increased significantly, and has maintained a long-term trend of growth, which means that the GRN has benefited larger group of patients and improved their accessibility. It is noteworthy that all PD-1 drug volumes declined by February 2022. However, this change was not caused by policy impacts. This change may be related to the cyclicity of drug procurement in Chinese public hospitals. Most hospitals in China do not make large-volume drug purchases in January and February, largely due to financial settlements, health insurance payment settlements, and other factors. But this impact is relatively small, so seasonality and cyclicality do not affect our main findings.

Furthermore, the findings demonstrate that the price reduction of the first-time GRN is larger than the second-time GRN for the same PD-1 drug, and the impact of the first GRN on the volume and expenditure is also greater. Camrelizumab received the most significant price reduction in the first-time GRN, resulting in a substantial increase in volume within one year of policy implementation. However, owing to the failure of Camrelizumab's negotiation in the second-time GRN and the influence of the inclusion of other PD-1 drug indications in the NRDL, there has been a downward trend in both the volume and expenditures of Camrelizumab after January 2022.

How to balance the cost of high-value innovative drugs with their clinical needs is a major global challenge. China has effectively reduced the price of PD-1 drugs through the GRN policy and improved patient affordability. This policy can help other health care systems optimise the reimbursement decisions of high-value innovative drugs. Nevertheless, the upward trend in expenditure on PD-1 drugs is becoming less pronounced due to the substantial reduction in the price of PD-1 drugs. From the trend of change after the second time of GRN in **Figure 2** and **Figure 3**, it reveals that the rising trend in expenditure for Toripalimab and Tislelizumab is smaller than the rising trend of their volume. Moreover, the expenditure of Sintilimab has declined despite an increase in its use. In 2022, the total income of China's basic medical insurance (including maternity insurance) fund was 309 221.17 billion CNY, and the total expenditure of the national basic medical insurance (including maternity insurance) fund was 245 972.24 billion CNY [36]. Despite the considerable income and expenditure of China's basic medical insurance fund, it is important to note that as of 2022, the number of participants in China's basic medical insurance was 1.346 billion people, with the participation rate is stable at more than 95%, and the per capita medical insurance fund is relatively small. Consequently, the formulation of future GRN policies should address the simultaneous objectives of ensuring the sustainability of the health insurance fund, meeting patients' fundamental pharmaceutical needs, and ensuring the profits of pharmaceutical manufacturers.

At the same time, policy-makers also need to consider the interests of pharmaceutical companies. For example, the manufacturer of Sintilimab sold a greater quantity of drugs after the second time of GRN, but the revenue it received was less than before. In order to ensure that pharmaceutical companies continue to invest in research and development (R&D), policy-makers need to consider and ensure reasonable profits for them. For example, in the GRN process, health policy-makers should balance affordability with R&D incentives. The government authorities can rigorously measure the R&D, production and operating costs to ensure that manufacturers still have a reasonable profit margin after deducting various costs. As GRN is carried out once a year, government authorities can establish communication mechanisms with drug manufacturers, keep abreast of their needs and difficulties, and adjust GRN policies to market changes. In addition, the health care department can cooperate with the finance department to provide tax incentives for PD-1drug manufacturers.

This result also offers some insights for other PD-1 drug manufacturers: in the event of a failed negotiation, whether their PD-1 drugs will be significantly influenced by the success of the negotiation of similar PD-1 drugs. In addition to the number of negotiations, the impact of the time sequence of inclusion in the medical insurance drug list should also be taken into consideration. **Figure 2** and **Figure 3** demonstrate that Sintilimab, which was the first PD-1 drug included in the NRDL after the successful negotiation, had a significantly higher volume and expenditure than the other PD-1 drugs. This advantage was maintained until the second time of GRN. This evidence demonstrates that the first PD-1 drug included in NRDL may gain more market share.

### Strengths and limitations

As far as we know, this is the first study to evaluate the impacts of GRN on PD-1 drug price, volume and procurement expenditure in China. Due to the short time development of GRN, there are a limited number of drugs that have conducted multiple-times GRN, leading to the lack of analysis on the impact of multiple-times GRN for the same type of drugs. A multiple-treatment period ITS was employed to evaluate the impact of multiple GRNs on the PD-1 drugs. The results of this study are of value to both health insurance policy-makers and PD-1 drug manufacturers.

Nonetheless, this study has the following limitations. First, the data used in this study were drug procurement data, which do not fully represent the actual number of drugs used in the clinic and are not known for the proportion of non-reimbursement indications in NRDL. Further studies using actual reimbursement data from the National Healthcare Security Administration are needed. Second, due to the absence of procurement data from community pharmacies in China, the results can only represent the impact of GRN for PD-1 drugs in hospitals. However, the majority drugs in China are sold in hospitals, especially for cancer drugs. Therefore, the data remain sufficiently representative. Third, due to some PD-1 drugs were not negotiated in the same year, there is some bias in the comparison between different PD-1 drugs. More importantly, the lack of clinical and patient-level data may reduce the generality of this study. These findings can only be used as a reference for health policy-makers, not for clinical decision-making. Further studies are needed to assess the actual financial burden that PD-1 drugs impose on patients, as well as the clinical benefit to patients after the GRN.

## CONCLUSIONS

China’s GRN policy has improved the affordability and accessibility of PD-1 drugs. However, some PD-1 drugs included in NRDL may significantly increase the expenditure of the health insurance fund. Those PD-1 drugs that are successfully negotiated first may gain a first-mover advantage. It is very important that policy-makers need to consider the reasonable interests of PD-1 drug manufacturers while ensuring the sustainability of the basic health insurance fund, in order to incentivise their R&D investment for innovative drugs.

## Additional material


Online Supplementary Document
