# Supplementary material for: Evaluation of changes in price, volume and expenditure of PD-1 drugs following the government reimbursement negotiation in China: a multiple-treatment period interrupted time series analysis
Source: J Glob Health. 2025 Apr 18;15:04069. doi: 10.7189/jogh.15.04069 (PMC12006830; doi:10.7189/jogh.15.04069)

Supplement to: Yi H, Cai M, Wei X, Cao Y, Kuai L, Xu D, Qiu Y, Han S. Evaluation of changes in price, volume and expenditure of PD-1 drugs following the government reimbursement negotiation in China: a multiple-treatment period interrupted time series analysis. J Glob Health. 2025;15:04069.

Table S1. Details of the first-time and second-time negotiations for four PD-1 drugs

**Drug name**	**Launch time ⁎**	**First procurement time recorded by CMEI †**	**Implementation of first negotiation**		**Implementation of second negotiation**	
			**time**	**Indications in NRDL**	**time**	**Indications in NRDL**
Toripalimab	December 2018	March 2019	March 2021	Treatment of unresectable or metastatic melanoma is limited to patients who have previously failed systemic therapy.	January 2022	1. This product is suitable for the treatment of unresectable or metastatic melanoma that has failed previous systemic treatment.<br>2. This product is suitable for the treatment of patients with recurrent/metastatic nasopharyngeal carcinoma who have failed to receive second-line or above systemic therapy.<br>3. This product is suitable for the treatment of locally advanced or metastatic urothelial carcinoma that has failed platinum-based chemotherapy including progression within 12 months after neoadjuvant or adjuvant chemotherapy.<br><br>The conditional approval in China was based on

Drug name	Launch time *	First procurement time recorded by CMEI †	Implementation of first negotiation		Implementation of second negotiation	
			time	Indications in NRDL	time	Indications in NRDL
						objective response rate results from a single-arm clinical trial. Full approval for this indication will depend on ongoing confirmatory clinical trials demonstrating long-term clinical benefit in Chinese patients.
Sintilimab	December 2018	March 2019	January 2020	Patients with relapsed or refractory classical Hodgkin's lymphoma after at least second-line systemic chemotherapy were limited.	January 2022	1. This product is suitable for the treatment of relapsed or refractory classical Hodgkin lymphoma after at least second-line systemic chemotherapy. This indication was given conditional approval based on objective response rate and response duration results from a single-arm clinical trial. Full approval for this indication will depend on ongoing confirmatory randomized, controlled clinical trials demonstrating significant clinical benefit with sintilizumab versus standard of care.<br>2. Sintilimab plus pemetrexed and platinum-based chemotherapy for the treatment of previously untreated advanced or recurrent epidermal growth factor receptor (EGFR) mutation-negative and anaplastic lymphoma kinase (ALK) -negative non-

Drug name	Launch time *	First procurement time recorded by CMEI †	Implementation of first negotiation		Implementation of second negotiation	
			time	Indications in NRDL	time	Indications in NRDL
						squamous non-small cell lung cancer.<br>3. Sintilimab plus gemcitabine and platinum-based chemotherapy for first-line treatment of unresectable advanced or recurrent squamous cell non-small-cell lung cancer.<br>4. Sintilimab plus bevacizumab for first-line treatment of unresectable or metastatic hepatocellular carcinoma not previously treated systemically
Camrelizumab	May 2019	August 2019	March 2021	1. Treatment of patients with relapsed or refractory classical Hodgkin lymphoma after at least second-line systemic chemotherapy.<br>2. Treatment of patients with advanced hepatocellular carcinoma previously treated with sorafenib and/or oxaliplatin-containing systemic chemotherapy.<br>The combination of pemetrexed and carboplatin is indicated for first-line treatment of epidermal growth factor receptor (EGFR) mutation-negative and	NA ‡	NA

Drug name	Launch time *	First procurement time recorded by CMEI †	Implementation of first negotiation		Implementation of second negotiation	
			time	Indications in NRDL	time	Indications in NRDL
				anaplastic lymphoma kinase (ALK) - negative, unresectable locally advanced or metastatic non-squamous non-small cell lung cancer (NSCLC).<br>4. Treatment of patients with locally advanced or metastatic esophageal squamous cell carcinoma with disease progression or intolerance after prior first-line chemotherapy.		
Tislelizumab	December 2019	March 2020	March 2021	Limited to recurrent or refractory patients with typical Hodgkin lymphoma treated with at least second-line chemotherapy; Failures of platinum-containing chemotherapy with high PD-L1 expression included treatment of locally advanced or metastatic urothelial carcinoma that progressed within 12 months of neoadjuvant or adjuvant chemotherapy.	January 2022	1. Classical Hodgkin's lymphoma: This product is suitable for the treatment of relapsed or refractory classical Hodgkin's lymphoma after at least second-line systemic chemotherapy. This indication was given conditional approval based on objective response rate and response duration results from a single-arm clinical trial. Full approval for this indication will depend on ongoing confirmatory randomized, controlled clinical trials demonstrating significant clinical benefit with this drug as compared with standard of care.

Drug name	Launch time *	First procurement time recorded by CMEI †	Implementation of first negotiation		Implementation of second negotiation	
			time	Indications in NRDL	time	Indications in NRDL
						2. Urothelial carcinoma: this product is suitable for the treatment of locally advanced or metastatic urothelial carcinoma with high PD-L1 expression and failure of platinum-based chemotherapy including progression within 12 months after neoadjuvant or adjuvant chemotherapy. This indication was given conditional approval based on objective response rate and response duration results from a single-arm clinical trial. Full approval for this indication will depend on ongoing confirmatory randomized, controlled clinical trials demonstrating significant clinical benefit with this drug as compared with standard of care.<br>3. Non-small cell lung cancer: combined with paclitaxel and carboplatin for the first-line treatment of unresectable locally advanced or metastatic squamous non-small cell lung cancer. It is used in combination with pemetrexed and platinum-based chemotherapy as the first-line treatment for epidermal growth factor receptor

Drug name	Launch time *	First procurement time recorded by CMEI †	Implementation of first negotiation		Implementation of second negotiation	
			time	Indications in NRDL	time	Indications in NRDL
						(EGFR) mutation-negative and anaplastic lymphoma kinase (ALK) -negative, unresectable locally advanced or metastatic non-squamous non-small cell lung cancer.<br>4. Hepatocellular carcinoma: This product is suitable for the treatment of hepatocellular carcinoma (HCC) after at least one systemic treatment. This indication was given conditional approval based on objective response rate and overall survival results from a phase II clinical trial. Full approval for this indication will depend on ongoing confirmatory randomized, controlled clinical trials demonstrating significant clinical benefit with this drug as compared with standard of care.

PD-1 - programmed death, CMEI - Chinese Medical Economic Information, NRDL - National Reimbursement Drug List.

* The launch time form the National Medical Products Administration (NMPA), meaning that the drug is approved at this time.

† After a drug is approved by the NMPA, it takes some time for it to enter the hospital drug list and be used in clinical practice, so the first purchase time may be delayed.

‡ Camrelizumab failed for the second-time negotiations.

Table S2. PD-1 drug prices before and after GRN implementation

Drug name	Price before the GRN implementation	Price after the first GRN implementation	Price after the second GRN implementation
Toripalimab	CNY￥7200 / 240 mg	CNY￥2100 / 240 mg	CNY￥1885 / 240 mg
Sintilimab	CNY￥7838 / 100 mg	CNY￥2843 / 100 mg	CNY￥1080 / 100 m
Camrelizumab	CNY￥19800 / 200mg	CNY￥2576.64 / 200mg	NA *
Tislelizumab	CNY￥10688 / 100mg	CNY￥2180 / 100mg	CNY￥1450 / 100mg

PD-1 - programmed death, CMEI - Chinese Medical Economic Information, NRDL - National Reimbursement Drug List, CNY – Chinese Yuan.

* Camrelizumab failed for the second-time negotiations.

Table S3. ITS statistical results of PD-1 drug prices

Outcome measures	PD-1 drugs	Coefficient	Estimate	Std. Error	P value	95% CI
Price (in DDDc)	Toripalimab	β_0	458.554	8.055	0	442.273 to 474.834
		β_1	-1.165	1.054	0.275	-3.295 to 0.964
		β_2	-304.415	18.871	0	-342.554 to -266.276
		β_3	6.988	3.751	0.07	-0.592 to 14.569
		β_4	-32.964	13.124	0.016	-59.487 to -6.44
		β_5	-5.735	3.127	0.074	-12.055 to 0.586
	Sintilimab	β_0	751.262	4.925	0	741.309 to 761.215
		β_1	-2.213	1.625	0.181	-5.497 to 1.072
		β_2	-455.628	12.459	0	-480.81 to -430.447
		β_3	1.879	1.63	0.256	-1.415 to 5.174
		β_4	-164.099	3.844	0	-171.867 to -156.331
		β_5	0.524	0.364	0.158	-0.212 to 1.259
	Camrelizumab	β_0	1441.545	24.569	0	1391.764 to 1491.326
		β_1	-4.792	4.089	0.249	-13.078 to 3.494
		β_2	-1151.75	50.728	0	-1254.534 to -1048.96
		β_3	5.52	4.441	0.222	-3.478 to 14.519
	Tislelizumab	β_0	1015.727	3.361	0	1008.842 to 1022.613
		β_1	-0.147	0.646	0.822	-1.469 to 1.176
		β_2	-809.9	5.266	0	-820.686 to -799.113
		β_3	0.226	1.365	0.87	-2.57 to 3.022
		β_4	-67.215	5.738	0	-78.969 to -55.461
		β_5	-0.029	1.021	0.978	-2.121 to 2.063

DDDc - cost per defined daily dose, PD - programmed death, CI - confidence interval.

Figure S1. Price changes for Toripalimab

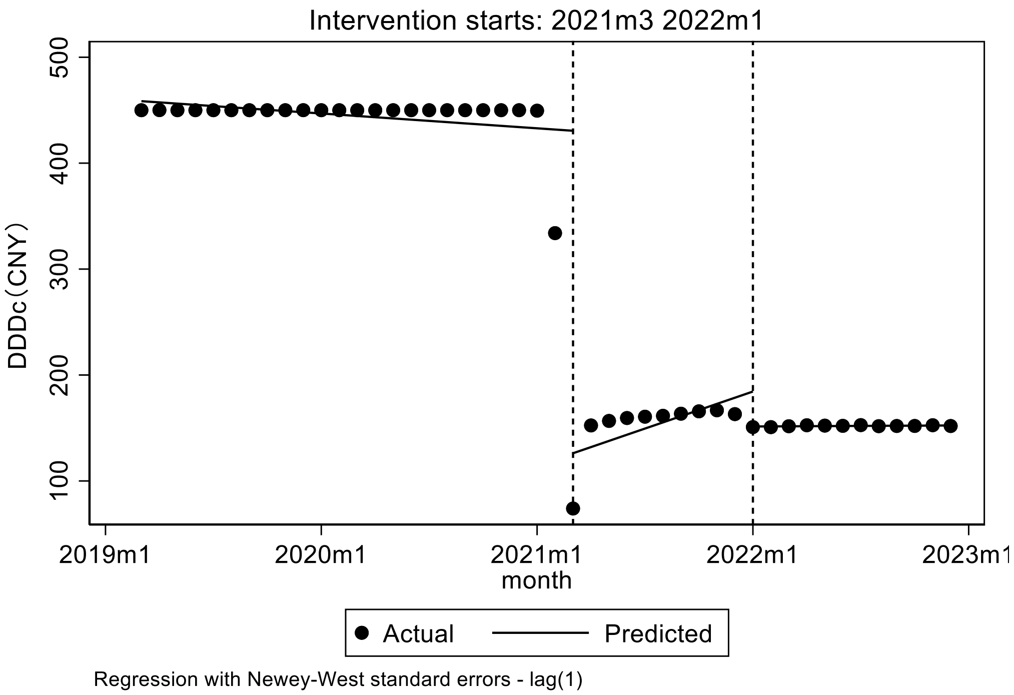

Figure S2. Price changes for Sintilimab

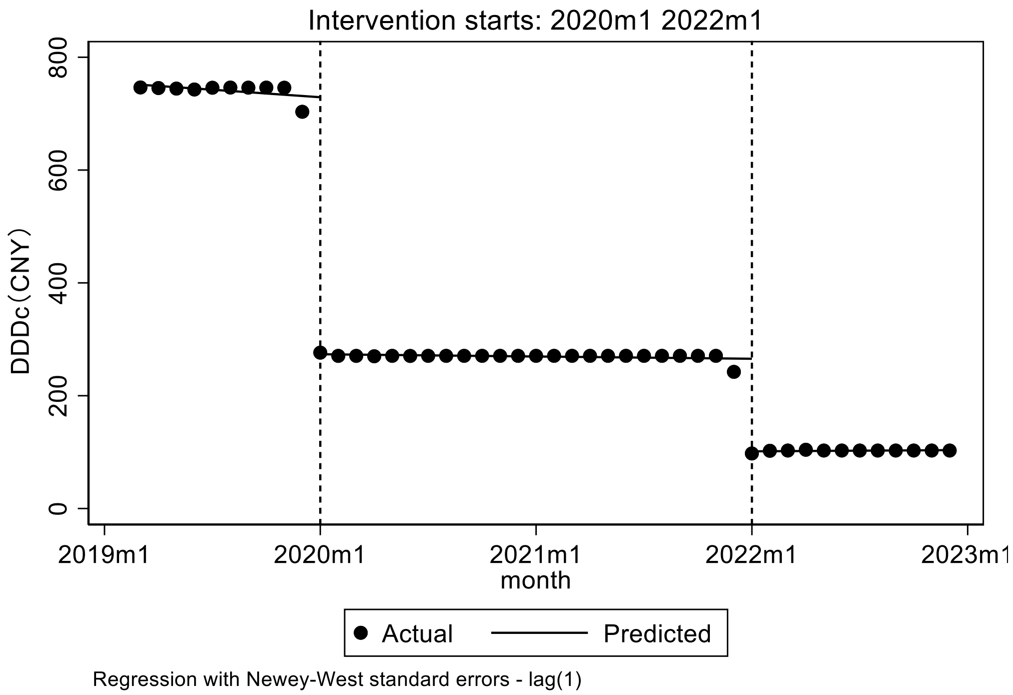

Figure S3. Price changes for Camrelizumab

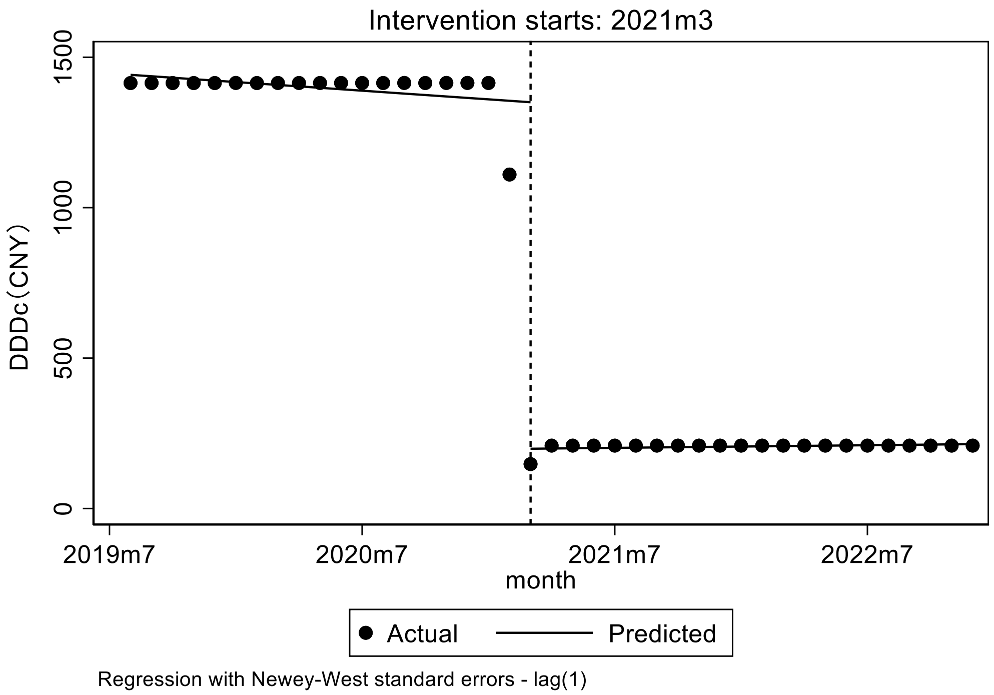

Figure S4. Price changes for Tislelizumab

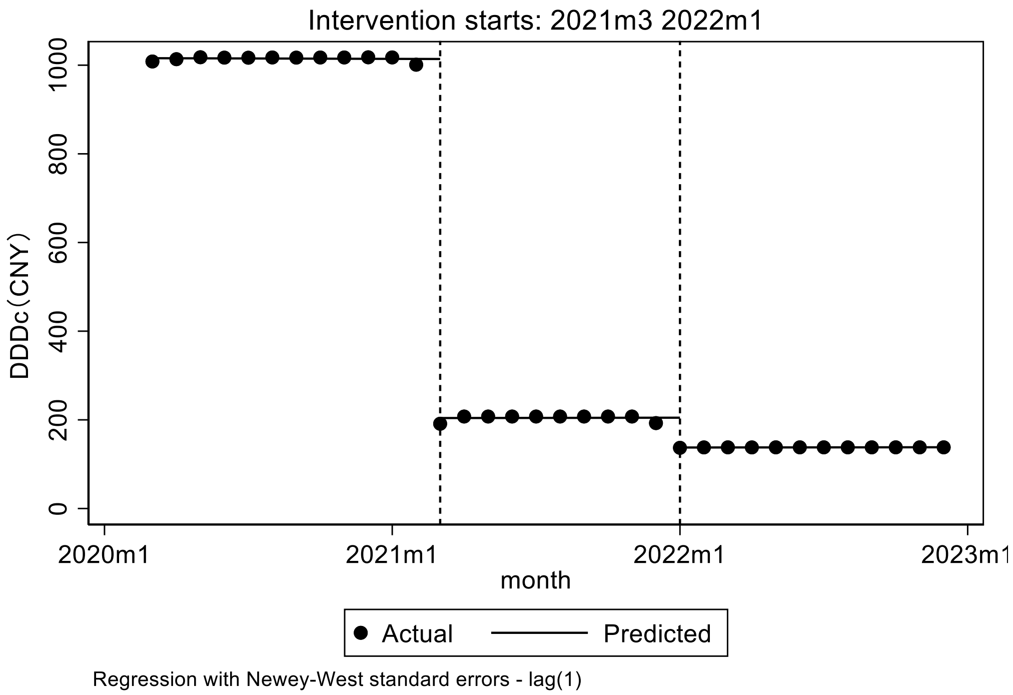

Table S4. ITS statistical results of PD-1 drug procurement volumes

Outcome measures	PD-1 drugs	Coefficient	Estimate	Std. Error	P value	95% CI
Volume (in DDDs)	Toripalimab	β_0	7.11	1.579	0	3.918 to 10.302
		β_1	0.666	0.151	0	0.362 to 0.971
		β_2	9.211	6.35	0.155	-3.623 to 22.045
		β_3	-0.27	0.715	0.707	-1.715 to 1.175
		β_4	5.282	5.452	0.338	-5.737 to 16.301
		β_5	2.847	1.084	0.012	0.655 to 5.038
	Sintilimab	β_0	-0.688	0.575	0.238	-1.85 to 0.474
		β_1	0.669	0.156	0	0.353 to 0.984
		β_2	18.017	7.324	0.018	3.214 to 32.819
		β_3	6.918	0.53	0	5.847 to 7.99
		β_4	67.944	28.246	0.021	10.856 to 125.032
		β_5	-2.363	4.88	0.631	-12.226 to 7.5
	Camrelizumab	β_0	-1.494	0.778	0.063	-3.07 to 0.083
		β_1	1.044	0.075	0	0.893 to 1.196
		β_2	159.549	19.953	0	119.12 to 199.979
		β_3	-2.942	1.377	0.039	-5.732 to -0.151
	Tislelizumab	β_0	-0.122	0.095	0.213	-0.317 to 0.074
		β_1	0.295	0.015	0	0.264 to 0.326
		β_2	23.837	6.597	0.001	10.324 to 37.35
		β_3	12.425	1.897	0	8.54 to 16.309
		β_4	102.185	26.52	0.001	47.862 to 156.509
		β_5	0.027	5.119	0.996	-10.458 to 10.512

DDDs - defined daily doses, PD - programmed death, CI - confidence interval.

Figure S5. Procurement volume changes for Toripalimab

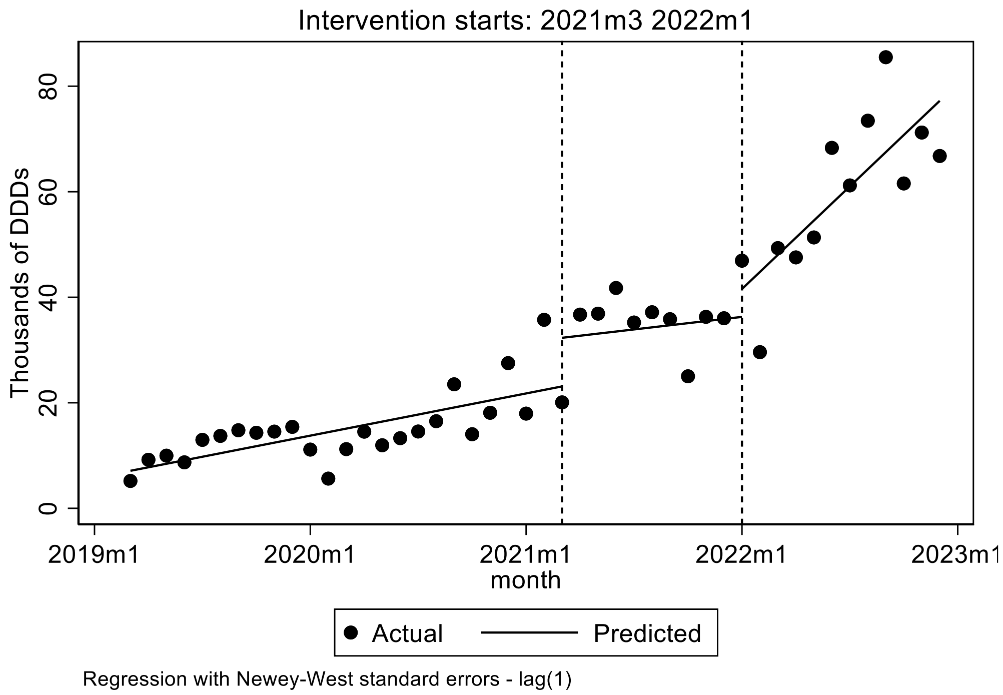

Figure S6. Procurement volume changes for Sintilimab

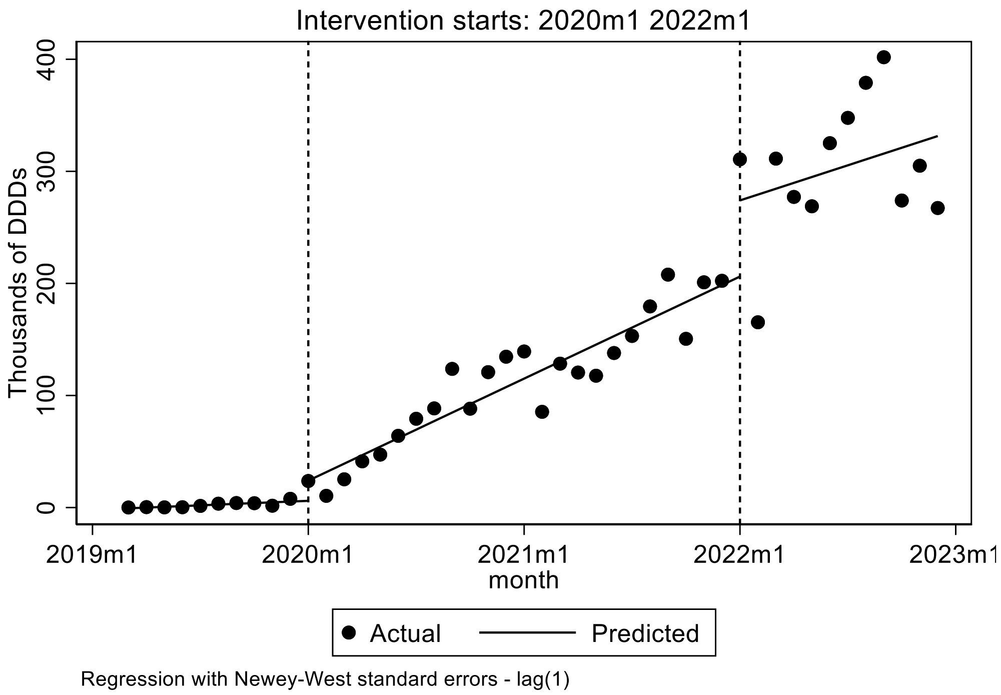

Figure S7. Procurement volume changes for Camrelizumab

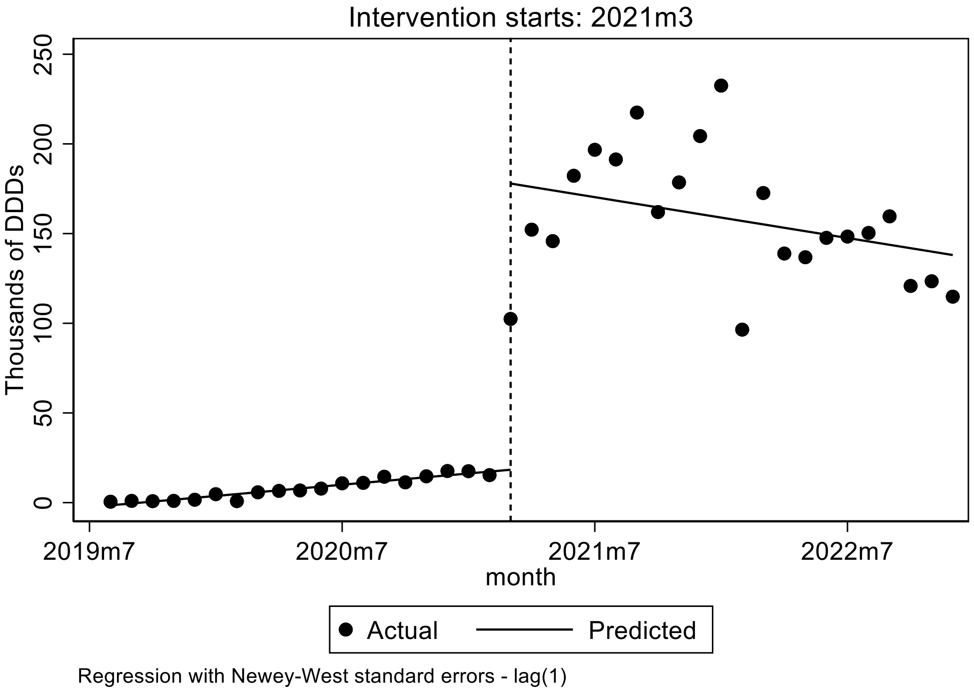

Figure S8. Procurement volume changes for Tislelizumab

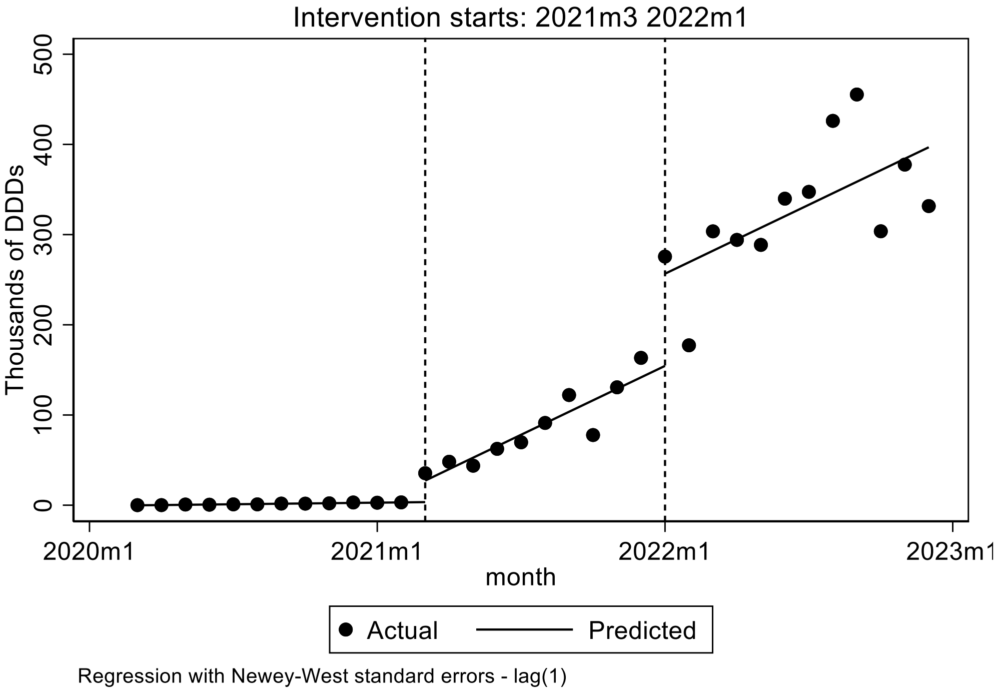

Table S5. ITS statistical results of PD-1 drug procurement expenditures

Outcome measures	PD-1 drugs	Coefficient	Estimate	Std. Error	P value	95% CI
Sending (million CNY)	Toripalimab	β_0	3.504	0.582	0	2.328 to 4.681
		β_1	0.258	0.045	0	0.168 to 0.348
		β_2	-5.28	1.522	0.001	-8.356 to -2.204
		β_3	-0.061	0.169	0.718	-0.404 to 0.281
		β_4	-0.098	0.997	0.922	-2.113 to 1.916
		β_5	0.3	0.21	0.162	-0.125 to 0.725
	Sintilimab	β_0	-0.465	0.399	0.251	-1.272 to 0.341
		β_1	0.481	0.107	0	0.265 to 0.696
		β_2	2.595	2.208	0.247	-1.867 to 7.057
		β_3	1.514	0.176	0	1.159 to 1.87
		β_4	-27.069	3.602	0	-34.35 to -19.789
		β_5	-1.4	0.521	0.01	-2.453 to -0.348
	Camrelizumab	β_0	-1.695	1.141	0.146	-4.007 to 0.618
		β_1	1.404	0.142	0	1.117 to 1.69
		β_2	11.172	4.698	0.023	1.653 to 20.692
		β_3	-1.726	0.403	0	-2.542 to -0.909
	Tislelizumab	β_0	-0.118	0.095	0.227	-0.313 to 0.077
		β_1	0.298	0.016	0	0.266 to 0.331
		β_2	2.345	1.035	0.031	0.224 to 4.466
		β_3	2.241	0.299	0	1.629 to 2.853
		β_4	4.119	3.87	0.296	-3.808 to 12.047
		β_5	-0.766	0.721	0.297	-2.242 to 0.71

CNY - Chinese Yuan, PD - programmed death, CI - confidence interval.

Figure S9. Procurement expenditure changes for Toripalimab

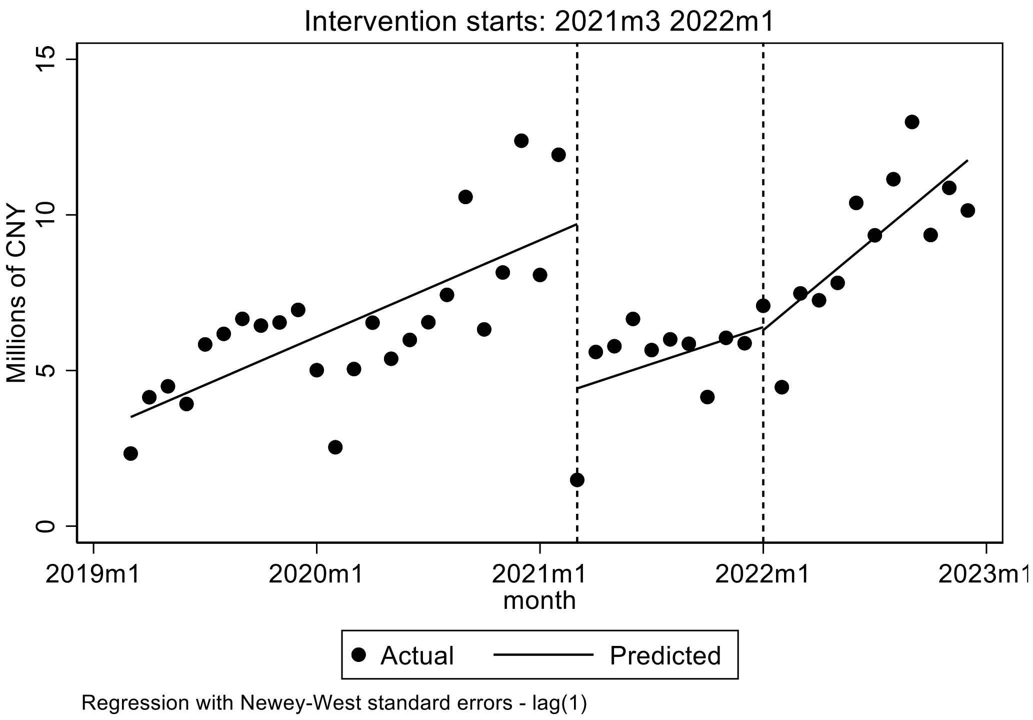

Figure S10. Procurement expenditure changes for Sintilimab

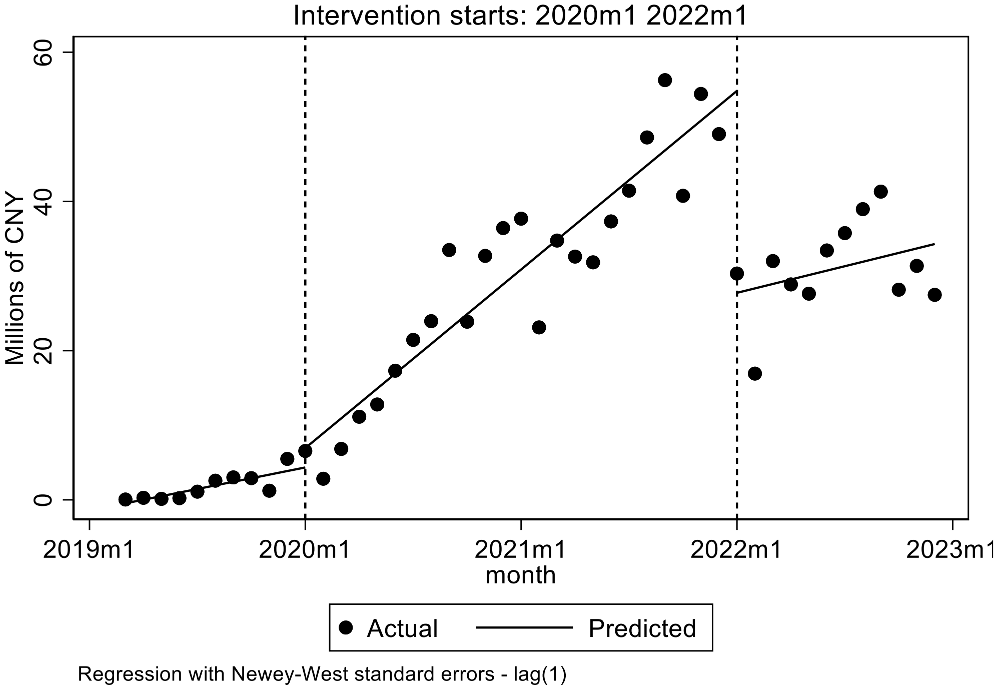

Figure S11. Procurement expenditure changes for Camrelizumab

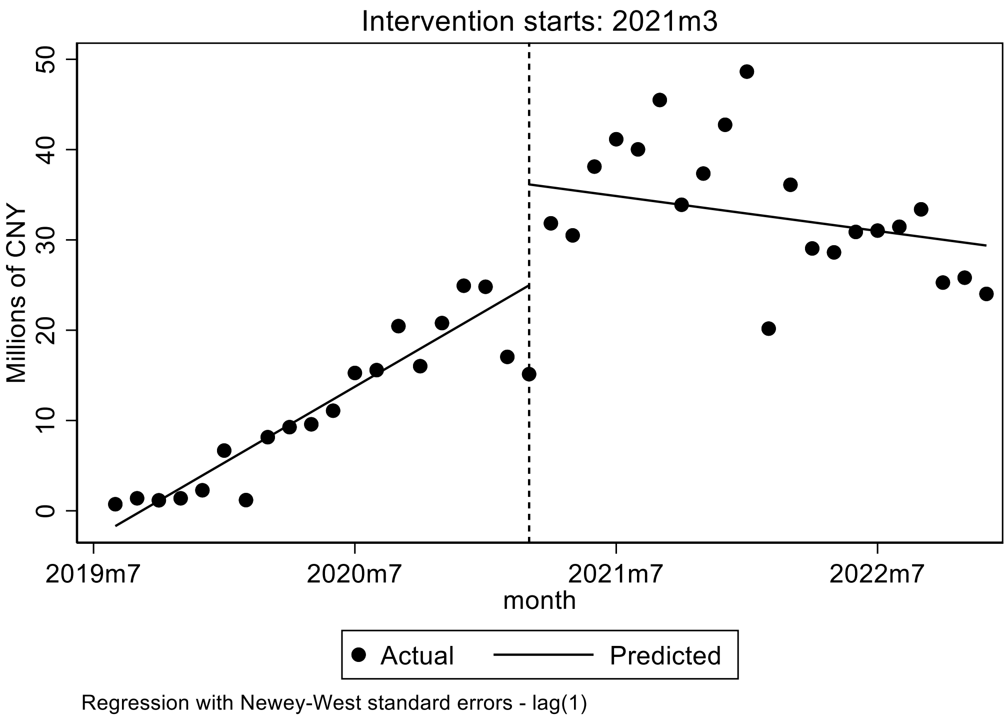

Figure S12. Procurement expenditure changes for Tislelizumab

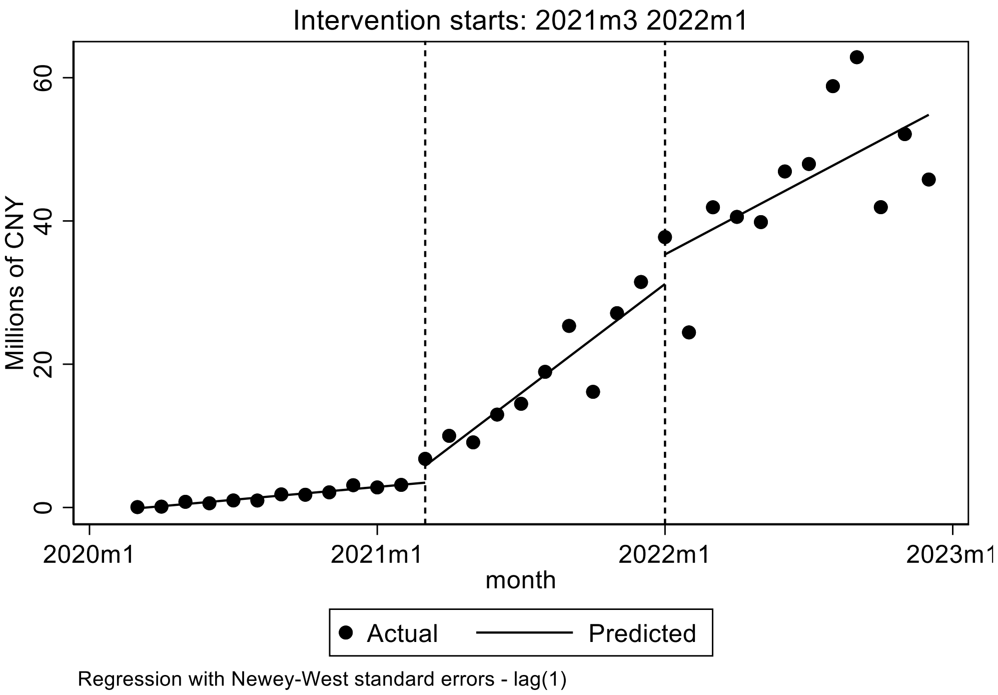

Supplement: Online Supplementary Document [file jogh-15-04069-s001.pdf]
